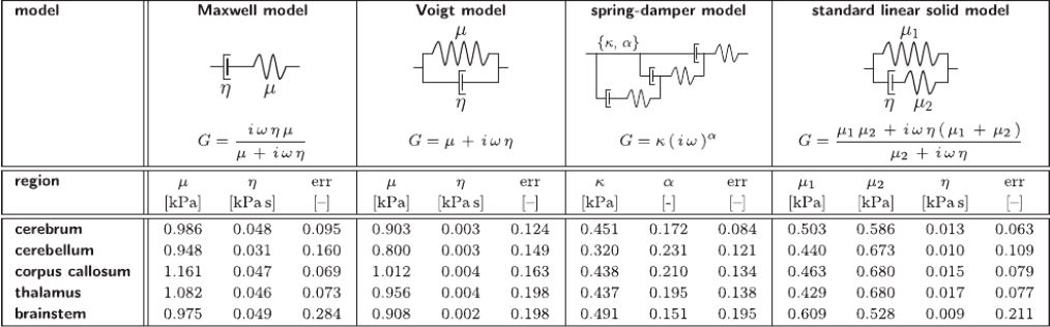# Brain stiffens post mortem

**DOI:** 10.1016/j.jmbbm.2018.04.009

**Published:** 2018-04-22

**Authors:** J. Weickenmeier, M. Kurt, E. Ozkaya, R. de Rooij, T.C. Ovaert, R.L. Ehman, K.Butts Pauly, E. Kuhl

**Affiliations:** a Department of Mechanical Engineering, Stanford University, Stanford, CA 94305, USA; b Department of Mechanical Engineering, Stevens Institute of Technology, Hoboken, NJ 07030, USA; c Department of Aerospace and Mechanical Engineering, University of Notre Dame, Notre Dame, IN 46556, USA; d Department of Radiology, Mayo Clinic, Rochester, MN 55905, USA; e Department of Radiology Stanford University Stanford, CA 94305, USA

**Keywords:** Magnetic resonance elastography, Brain, Rheology, Stiffening, In vivo

## Abstract

Alterations in brain rheology are increasingly recognized as a diagnostic marker for various neurological conditions. Magnetic resonance elastography now allows us to assess brain rheology repeatably, reproducibly, and non-invasively in vivo. Recent elastography studies suggest that brain stiffness decreases one percent per year during normal aging, and is significantly reduced in Alzheimer’s disease and multiple sclerosis. While existing studies successfully compare brain stiffnesses across different populations, they fail to provide insight into changes within the same brain. Here we characterize rheological alterations in one and the same brain under extreme metabolic changes: alive and dead. Strikingly, the storage and loss moduli of the cerebrum increased by 26% and 60% within only three minutes post mortem and continued to increase by 40% and 103% within 45 minutes. Immediate post mortem stiffening displayed pronounced regional variations; it was largest in the corpus callosum and smallest in the brainstem. We postulate that post mortem stiffening is a manifestation of alterations in polarization, oxidation, perfusion, and metabolism immediately after death. Our results suggest that the stiffness of our brain–unlike any other organ–is a dynamic property that is highly sensitive to the metabolic environment Our findings emphasize the importance of characterizing brain tissue in vivo and question the relevance of ex vivo brain tissue testing as a whole. Knowing the true stiffness of the living brain has important consequences in diagnosing neurological conditions, planning neurosurgical procedures, and modeling the brain’s response to high impact loading.

## 1. Introduction

With less than 3% of our body weight, our brain consumes 15% of cardiac output, 20% of oxygen, and 25% of total body glucose (Mergenthaler et al., 2013). It seems intuitive that our brain, more than any other organ, is highly sensitive to alterations in its biochemical environment Here we probe the brain’s sensitivity to biochemical alterations and expose a single brain to the most extreme change in metabolic conditions, from alive to dead. We characterize changes in brain rheology within three minutes post mortem using magnetic resonance elastography (Muthupillai et al., 1995), a rapidly developing technology that allows us to quantify the viscoelasticity of the living brain, repeatably, reproducibly, and non-invasively in vivo and in situ (Muthupillai and Ehman, 1996). Magnetic resonance elastography generates shear waves in soft tissues, images wave propagation, and correlates wave propagation to tissue stiffness in the form of elastograms (Kruse et al., 2000). While the technology was initially developed to identify regional stiffness variations and detect tumors in breast, liver, kidney, and prostate cancer (Mariappan et al., 2010), it is now widely used to characterize stiffness profiles across various living tissues including the breast, the heart, kidneys, lung, skeletal muscle, and the brain (Glaser et al., 2012). With increasing confidence in the method itself, elastography is emerging as a diagnostic biomarker for various neurological conditions (Hiscox et al., 2016). Recent studies have reported an annual stiffness decay of 0.8% during normal aging (Sack et al., 2009), and a stiffness reduction of 20% in chronic progressive multiple sclerosis (Enzinger et al., 2015) and 8% in Alzheimer’s disease (Murphy et al., 2011). Its inherent high regional specificity makes elastography uniquely suited to differentiate between specific subtypes of dementia including Alzheimer’s disease, frontotemporal dementia, normal pressure hydrocephalus, and dementia with Lewy bodies (ElSheikh et al., 2017).

Overwhelming evidence suggests that elastography can quantify significant differences between certain subject groups (Sack et al., 2011; Streitberger et al., 2012; Arvin et al., 2015; Murphy et al., 2016). However, to serve as a truly diagnostic tool, the technology seems to lack a thorough verification and validation. Elastography recordings depend on multiple factors including the method to generate shear waves (Badachhape et al., 2017), the selection of the actuation frequency (Hiscox et al., 2016), the inversion technique to extract the dynamic moduli (Johnson and Telzer, 2017), and, more recently, the analysis of physiological vibration (Zorgani et al., 2015). Few studies have calibrated their elastography measurements against phantom gels with known properties (Atay et al., 2008; Kruse et al., 2008) or against finite element simulations (Miller et al., 2000; Bayly et al., 2012). Yet, to date, there is no consistent comparison of in vivo and in situ elastography recordings with ex vivo measurements on one and the same sample. The only study that directly compared elastography to standard mechanical testing compared ex vivo elastography to ex vivo rheometry on explanted brains at frequencies that differed by two orders of magnitude (Vappou et al., 2008). The only study that compared same-sample measurements in vivo and in situ perturbed the natural environment by removing the skull to access the brain for indentation testing (Gefen and Margulies, 2004).

Comparing in vivo recordings to ex vivo measurements seems to be critical to correctly interpret the ex vivo stiffness values of brain tissue that have been recorded and reported for more than half a century (Chatelin et al., 2010). Despite comprehensive efforts to characterize the rheology of the brain ex vivo (Franze et al., 2013), the literature continues to provide contradictory results with respect to tissue anisotropy (Feng et al., 2013a; Budday et al., 2015), regional variations (Prange and Margulies, 2002; Forte et al., 2017), gray and white matter stiffness ratios (Christ et al., 2010; Weickenmeier et al., 2016), and stiffness alterations post mortem (Budday et al., 2015; Nicolle et al., 2004). These ongoing controversies point towards an urgent need to better understand the mechanistic origin of measurement discrepancies. Knowing the in vivo stiffness of the living brain has important consequences for understanding brain form, function, and failure (Goriely et al., 2015). The stiffness is the most critical input parameter for various computational brain models to predict safety level thresholds (Qoots et al., 2013), simulate surgical procedures (Goriely et al., 2016), model blast and impact situations (Cotton et al., 2016), and design protective devices (Nyein et al., 2010).

Here we compare the in vivo, in situ, and ex vivo rheology of one and the same brain under similar dynamic conditions. We recorded the region-specific in vivo and in situ brain viscoelasticity using elastography at five activation frequencies, 40, 60,80, 90, and 100 Hz, in five distinct regions of the brain, the cerebrum, cerebellum, corpus callosum, thalamus, and brainstem, and compared the results to the ex vivo viscoelasticity from nanoindentation in the same frequency regime. In addition to the mechanical characterization, we performed a histochemical analysis to reveal a mechanistic correlation between stiffness and myelin density. Finally, we converted our recordings to the region-specific parameters of four widely used viscoelastic models, the Maxwell, Voigt, spring-damper, and standard linear solid models.

## 2. Materials and methods

This study was approved by the Research Compliance Office at Standford University. It complies with IRB and Animal Care and Use guidelines and was conducted according to Standford University policy. To characterize regional mechanical and microstructural changes in the living and dead porcine brain–in vivo–we combined magnetic resonance elastography, dynamic nanoindentation, and a histochemical analysis.

### Animal preparation

Our study is based on one and the same brain of a 50.2 kg female Yorkshire pig. Prior to scanning, we sedated the animal with 6 mgAg intramuscular tiletamine zolazepam (Telazol, Fort Dodge, IA), which we induced with 3% isoflurane (Henry Schein Animal Health Dublin, OH) in oxygen delivered by a face mask. We intubated the trachea with an endotracheal tube, 8 mm in diameter, and maintained anesthesia with 1% to 3% isoflurane in oxygen using mechanical ventilation. To administer drugs and monitor the systemic arterial blood pressure, we placed percutaneous catheters in the left femoral vein and artery. We monitored heart rate, electrocardiogram, pulse oximetry, and end-tidal C02 throughout the imaging sequence. Throughout anesthesia, we intravenously administered lactated Ringer’s solution (Abbott Laboratories, Deerfield, IL) at a rate of 10 to 15 mL/kg per hour to compensate for fluid loss. We performed magnetic resonance elastography in vivo, then euthanized the animal with Euthanasia Solution (Vedco, Inc., St Joseph, MO), confirmed death by an electrocardiogram and auscultation, placed the animal back into the scanner, and performed post mortem magnetic resonance elastography in situ.

### Magnetic resonance elastography

For magnetic resonance elastography, we used the 3T research scanner (GE Healthcare, Waukesha, WI) at the Richard M. Lucas Center for Imaging at Stanford University following our established imaging protocols (Weickenmeier et al., 2018). To accommodate the full pig head and activation pillow, we used a single-channel split head coil and positioned the animal in the supine position for optimal transmission of the mechanical activation. We placed the acoustic passive actuator (Mayo Clinic, Rochester, MN) underneath the pig’s head and connected the internal activation pillow to an external acoustic active actuator to generate continuous vibrations at a selected frequency (Kruse et al., 2008). These vibrations transmit through the skull and induce shear waves inside the brain. We used three-dimensional magnetic resonance elastography and imaged the wave field using three directions of motion encoding at multiple time points in the wave cycle. We acquired the three-dimensional data with a 48-slice two-dimensional spin echoplanar imaging pulse sequence in the axial plane, at a resolution of 96 × 96, two shots, at a repetition time of TR= 2000 ms, an echo time of TE = 60 ms, and a field of view of FOV = 24 cm, using an array spatial sensitivity encoding technique, and applied a full three-dimensional inversion algorithm (Murphy et al., 2017). To ensure a synchronized receiver signal, we set the frequency of the motion encoding gradient equal to the actuation frequency. We acquired phase images at eight offset points sampled over one period of motion, and determined the elastograms from these images using three-dimensional direct inversion (Murphy et al., 2013; Manduca et al., 2001). For each actuation frequency; *ω*, we determine the isotropic storage and loss moduli *G′(ω)* and *G”*(*ω*) and map the frequency-dependent dynamic shear modulus *G* (*ω*),

$$G(\omega )=G^{\prime }(\omega )+i\;G^{\prime \prime }(\omega ),$$

and the effective shear stiffness *G*^eff^ (*ω)*,

$$G^{\text{eff}}(\omega )={\left[G^{\prime }{\left(\omega \right)}^{2}+G^{\prime \prime }{\left(\omega \right)}^{2}\right]}^{1/2}.$$

In total, we performed a structural Tl-weighted scan, in vivo elastography at five frequencies, 40, 60, 80, 90, and 100 Hz, and in situ elastography three and 45 min post mortem at a frequency of 80 Hz.

### Region-specific moduli

**Fig. 1.**
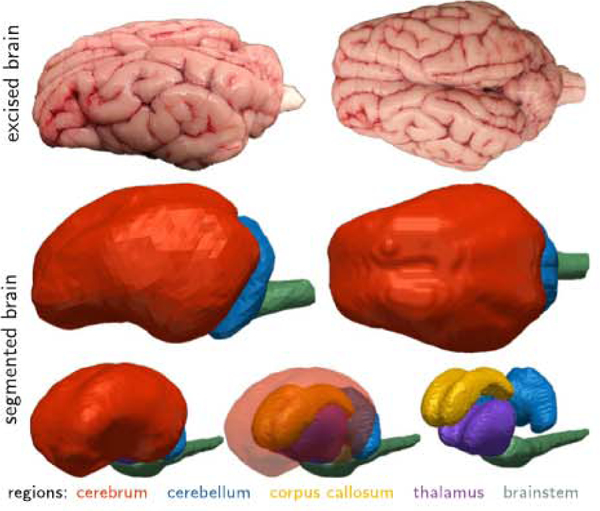
We characterized the in vivo, in situ, and ex vivo viscoelastic properties of one and the same brain using magnetic resonance elastography, nanoindentation, and histochemical analysis. To identify regional variations, we created volumetric models of the cerebrum (red), cerebellum (blue), corpus callosum (yellow), thalamus (purple), and brainstem (green), registered the storage and loss moduli across all five regions, and compared them against the viscoelastic properties from nanoindentation.

To assign the elastography measurements to specific brain regions, we performed a semi-automatic segmentation from Tl-weighted magnetic resonance images using Simpleware (Synopsys, Mountain View, USA). To identify individual brain regions, we combined initial greyscale thresholding, manual segmentation, and volumetric Gaussian smoothing and approximated the convoluted cortex by a smoothed surface. Fig. 1 shows the resulting cerebrum in red, the cerebellum in blue, the corpus callosum in yellow, the thalamus in purple, and the brainstem in green. For each substructure, we generated a binary mask to automatically analyze the elastography measurements and determine regional viscoelastic properties. We utilized the SPM12 software package for elastography image post-processing (Friston et al., 2007) and masked and registered the elastography images with the Tl-weighted images. We determined the transformation matrix that maps the elastography images to the Tl-weighted domain, and applied it to our storage, loss, and stiffness elastograms through a nonlinear registration procedure (Klein et al., 2009). To ensure a match with the three-dimensional Tl-weighted image domain, we performed interpolation-based re-slicing of our two-dimensional elastograms. Then we multiplied our binary mask and each elastogram to obtain the storage modulus, loss modulus, and effective stiffness for every voxel within each subregion’s segmentation. We report the moduli of each scan and region as boxplots with median, first and third quartile, minimum and maximum values, and data points above or below three-times the interquartile range. Each pixel of the elastography image stack that lies within the segmentation of each subregion resembles one data point in the boxplot.

### Nanoindentation

For nanoindentation, we excised the brain, stored it at 4 °C in phosphate-buffered saline solution, prepared three 5 mm-thick coronal slices from the anterior, mid, and posterior brain, placed each slice in a petri dish, and followed our established protocols for testing (Budday et al., 2015). We tested the samples using a Hysitron TI 950 Tri-boIndenterTM (Hysitron Inc., Eden Prairie, MN) with an xZ 500 extended displacement stage that allows for indenter tip displacements of up to 500 *μm* with a 1 nm displacement control precision and a force resolution of <0.1 nN (Weickenmeier et al., 2016). To minimize adhesion and prevent specimen dehydration, we stabilized each indentation region with a 12mm-diameter stainless steel washer and hydrated the tissue surface inside the washer with phosphate-buffered saline solution (Budday et al., 2015). We performed all measurements at room temperature, under displacement control, using a custom-made 1.5 mm diameter flat punch indenter (Weickenmeier et al., 2017). At 18 locations, ten in white matter and eight in gray matter, we performed a quasi-static indentation test with a tip displacement of 400 μm. After unloading, we performed a dynamic frequency sweep at ten frequencies ranging from 10 to 100 Hz at a penetration depth of 350 μm with the maximum amplitude of the device, 20 nm. We determine the elastic shear modulus *G* from the quasistatic contact stiffness *k*_s_, the mean slope of the loading curve at the indentation depth of the dynamic tests,

$$G=\frac{1-v^{2}}{2[1+v]}\frac{k_{\text{s}}\sqrt{\pi }}{2\sqrt{A}}=\frac{k_{\text{s}}}{4d}.$$

We determine the frequency-dependent storage and loss moduli *G*′(*ω*) and *G*”(*ω*) from the dynamic contact stiffness *k*_d_ and the damping *ωC*_d_,

$$G^{\prime }(\omega )=\frac{1}{2[1+\nu ]}\frac{k_{\text{d}}\sqrt{\pi }}{2\sqrt{A}}=\frac{k_{\text{d}}}{3\;d}$$

$$G^{\prime \prime }(\omega )=\frac{1}{2[1+v]}\frac{\omega C_{\text{d}}\sqrt{\pi }}{2\sqrt{A}}=\frac{\omega C_{\text{d}}}{3\;d}.$$

We assume tissue incompressibility, *v =* 0.5, and a contact area of *A = π d*^2^/4 for the flat punch indenter with diameter *d.*

### Histochemical analysis

Following mechanical testing, we fixed our coronal slices using 10% buffered formalin solution. We cut 25 × 25mm^2^ samples from the corona radiata of the left and right hemispheres of each slice as indicated by gray boxes in Fig. 6. We gradually dehydrated the samples by replacing tissue water by alcohol, cleared the samples by replacing alcohol by xylene, and embedded the dehydrated samples in paraffin wax blocks. From these, we cut 8–10 nm thick histological slices using a microtome. We stained the slices with a combined hematoxylin and eosin and luxol fast blue stain. Hematoxylin colors cell nuclei in blue, counter-staining with eosin colors eosinophilic intracellular and extracellular protein structures in shades of red, pink, and orange, and luxol fast blue colors myelin sheaths in blue, neuropil in pink, and nerve cells in purple. We used image processing and separated the blue myelin regions from the remaining microglial regions to determine the local myelin fraction as the ratio between the number of blue pixels over the total number of pixels, see Fig. 6.

### Viscoelastic modeling

To translate the frequency-dependent storage and loss moduli *G*′(*ω*) and *G*′(*ω*) into frequency-independent viscoelastic material parameters, we considered four frequently used viscoelastic models, the Maxwell model, Voigt model, spring-damper model, and standard linear solid model. Table 2 summarizes the characteristic rheological arrangement of springs and dampers and the dynamic shear moduli, $G(\omega )=G^{\prime }(\omega )+i\;G^{\prime \prime }(\omega ),$ for all four models. The dynamic modulus is G=(iωημ)/(μ+iωη) for the Maxwell model, G=μ=iωη for the Voigt model, $G=\kappa {\left(i\;\omega \right)}^{\alpha }$ for the spring-damper model, and $G=\left(\mu_{1}\mu_{2}+i\;\omega \eta \left(\mu_{1}+\mu_{2}\right)\right)/\left(\mu_{2}+i\;\omega \eta \right)$ for the standard linear solid model. We identified the model parameters, the shear stiffness *μ* [kPa], the viscosity *η* [kPa s], and the shear stiffness *κ* [kPa] with exponents *α* [-] using the Nelder-Mead method. Specifically, we minimized the normalized root mean square error between the experimental observations and model-based predictions of the storage and loss moduli at all *n*_*ω*_ = 5 frequencies of 40, 60, 80, 90, and 100 Hz,

$$\Phi =\frac{1}{2\;n_{\omega }}\sqrt{\sum_{i=1}^{n_{\omega }}{\left(\frac{G_{i}^{\prime \exp}-G_{i}^{\prime \text{mod}}}{G_{i}^{\prime }{}^{\exp}}\right)}^{2}+{\left(\frac{G_{i}^{\prime \prime }{}^{\exp}-G_{i}^{\prime \prime }{\;}^{\mathrm{mod}}\;}{G_{i}^{\prime \prime }{}^{\exp}}\right)}^{2}}.$$

To probe the robustness of the parameter identification, we performed 20,250 optimizations with varying initial conditions. We screened the physically-motivated parameter space by varying the initial values for the shear stiffness within *μ =* 0.1 – 4.0 kPa and the viscosity within *η* = 0.01 – 1.00 kPa s. While the parameter identifications of the Maxwell, Voigt, and spring-damper models were robust and insensitive to the initial conditions, the standard linear solid model presented multiple challenges: The low number of experimental measurement points, especially at low frequencies, and the lack of coupling between the shear stiffness *μ*_1_ and the loss modulus *G”,* generate strong sensitivity with respect to the initial conditions. To acknowledge this sensitivity, we report the five parameter sets with the lowest error values.

## 3. Results

### In vivo storage and loss moduli display regional variations

**Fig. 2.**
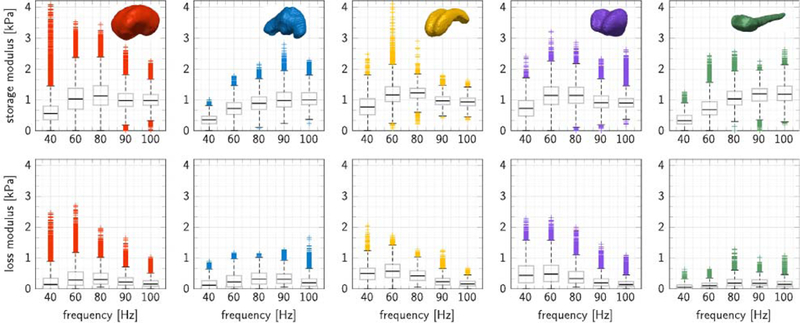
In vivo storage and loss moduli for five brain regions at five activation frequencies. Peak storage and loss moduli were 1.13 and 0.30 kPa for the cerebrum (red), 1.00 and 0.32 kPa for the cerebellum (blue), 1.22 and 0.57 kPa for the corpus callosum (yellow), 1.14 and 0.48 kPa for the thalamus (purple), and 1.19 and 0.19 kPa for the brainstem (green). Storage moduli increase with increasing frequency] loss moduli peaks at an intermediate frequency. Each graph highlights the median, first and third quartile, minimum and maximum values, and the measurement points above or below three-times the interquartile range.

Fig. 2 shows the in vivo storage and loss moduli of five regions of the brain, the cerebrum, cerebellum, corpus callosum, thalamus, and brainstem, recorded at five different actuation frequencies, 40, 50, 60, 80, 90, and 100 Hz. The graphs highlight the median, first and third quartile, minimum and maximum values, and measurement points above or below three-times the interquartile range. Each data point represents a pixel from the magnetic resonance elastography image stack. We observe a pronounced regional variation in dynamic moduli: peak storage moduli varied from 1.22 kPa in the corpus callosum, 1.19 kPa in the brainstem, 1.14 kPa in the thalamus, and 1.13 kPa in the cerebrum to 1.00 kPa in the cerebellum; peak loss moduli varied from 0.57 kPa in the corpus callosum, 0.48 kPa in the thalamus, 0.32 kPa in the cerebellum, and 0.30 kPa in the cerebrum to 0.19 kPa in the brainstem. We also observe a strong frequency dependency of both storage and loss moduli with storage moduli increasing with increasing frequency, and loss moduli peaking at intermediate frequencies. Of all five regions, the cerebrum displays most measurement points outside the three interquartile range. The cerebrum is the largest and most heterogeneous region. It includes internal interfaces with the falx and external interfaces with the ventricles where reflective shear waves can cause measurement errors and artifacts.

### In situ storage and loss moduli increase post mortem

**Fig. 3.**
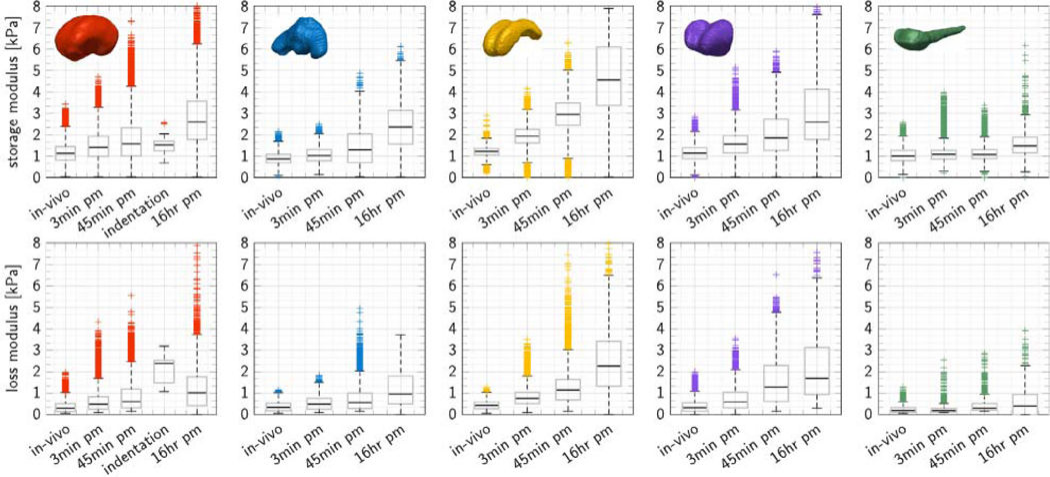
In vivo, in situ, and ex vivo storage and loss moduli for five brain regions at an activation frequency of 80 Hz. Storage and loss moduli increase with post mortem time. In vivo moduli are smallest in the living brain, followed by in situ moduli 3 and 45 min post mortem, and, for comparison, 16 h post mortem, measured in a different animal. Ex vivo moduli 16 h post mortem, measured on coronal slices of the same brain, are similar to in situ moduli 45 min post mortem. Stiffening within 45 min post mortem was 147% in the corpus callosum (yellow), followed by 61% in the thalamus (purple), 47% in the cerebellum (blue), 40% in the cerebrum (red), and 7% in the brainstem (green). Each graph highlights the median, first and third quartile, minimum and maximum values, and the measurement points above or below three-times the interquartile range.

Fig. 3 shows the in vivo, in situ, and ex vivo storage and loss moduli of five regions of the same brain, the cerebrum, cerebellum, corpus callosum, thalamus, and brainstem, recorded at an actuation frequency of 80 Hz. In situ recordings include magnetic resonance elastography measurements in vivo and 3 and 45 min post mortem on one and the same brain, and, for reference, measurements in a different animal 16 h post mortem (Weickenmeier et al., 2018). Ex vivo recordings include nanoindentation measurements 16 h post mortem on coronal slices of the initial brain. Storage and loss moduli increase markedly with post mortem time. Of all five regions, the corpus callosum stiffened most and the brainstem stiffened least Notably, within only 3 min post mortem, we observed stiffening of 58% in the corpus callosum, followed by 38% in the thalamus, 26% in the cerebrum, 17% in the cerebellum, and 8% in the brainstem. Stiffening continued within 45 min post mortem with 147% in the corpus callosum, followed by 61% in the thalamus, 47% in the cerebellum, 40% in the cerebrum, and 7% in the brainstem.

Table 1 summarizes the in vivo, in situ, and ex vivo storage and loss moduli of the brain, the cerebrum, cerebellum, corpus callosum, thalamus, and brainstem, recorded at an actuation frequency of 80 Hz. For the cerebrum, which includes both gray and white matter tissue, the storage and loss moduli increased 26% and 60% within 3 min post mortem and 40% and 103% within 45 min post mortem, compared to an increase of 35% and 687% observed in ex vivo slices of the same brain tested 16 h post mortem, but smaller than the in situ stiffening in another comparison porcine brain of 130% and 240% within 16 h post mortem. Most notably, the trend of post mortem stiffening was universal across all five regions. Storage and loss moduli increased the most in the corpus callosum with 142% and 171% and the least in the brainstem with 7% and 50%. Strikingly, at any given time point, irrespective of the post mortem time, the corpus callosum always remained the stiffest structure and the cerebellum and the brainstem remained the softest This suggests that *absolute stiffness* might increase considerably between the physiologically relevant in vivo setting and the mechanically accessible ex vivo setting; yet, the *relative stiffness* between the relevant regions of the brain seems to be relatively well preserved.

### Ex vivo storage and loss moduli are similar to in vivo moduli

**Fig. 4.**
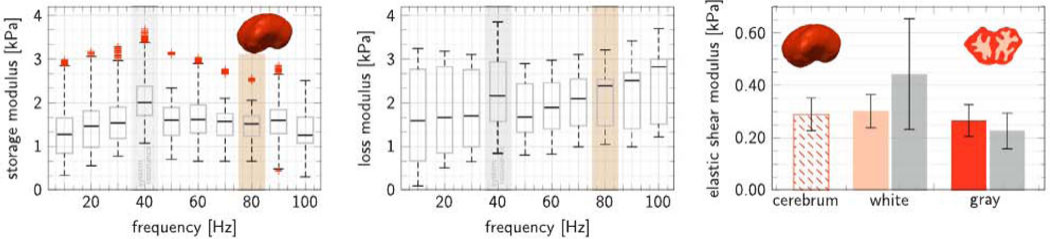
Ex vivo storage and loss moduli at ten activation frequencies and elastic shear modulus for white and gray matter regions. Storage moduli varied between 1.28 and 1.60 kPa; loss moduli varied between 1.59 and 2.83 kPa. Gray boxes at 40 Hz indicate the hardware resonance frequency; red boxes at 80 Hz indicate the moduli that we compare to the in vivo data. Elastic shear moduli varied between 0.29 ± 0.06 kPa in the cerebrum, 0.30 ± 0.07 kPa in white matter (orange), and 0.27 ± 0.06 kPa in gray matter (red). Gray boxes, for comparison, indicate measurements from our previous studies (Weickenmeier et al., 2016). Each graph highlights the median, first and third quartile, minimum and maximum values, and the measurement points above or below three-times the interquartile range.

Fig. 4 summarizes the ex vivo storage and loss moduli at ten activation frequencies, from 10 to 100 Hz, and the elastic shear modulus for white and gray matter regions of the cerebrum. We performed all dynamic and static measurements within 16 h post mortem using nanoindentation and averaged the recordings over n = 18 white and n = 8 gray matter regions. The gray boxes at 40 Hz indicate the hard-ware resonance frequency. The red boxes at 80 Hz indicate the storage and loss moduli of 1.52 kPa and 2.36 kPa that we compare to the in vivo data in Table 1. Each graph highlights the median, first and third quartile, minimum and maximum values, and the measurement points above or below three-times the interquartile range. The recorded storage moduli varied between 1.28 and 1.60 kPa and the loss moduli varied between 1.59 and 2.83 kPa. Quasi-static indentation recordings in different regions of the brain revealed that white matter was 13.5% stiffer than gray matter: The elastic shear modulus was 0.29 ± 0.06 kPa in the overall cerebrum, 0.30 ± 0.07 kPa in white matter, and 0.27 ± 0.06 kPa in gray matter. For comparison, these values agree well with our previous measurements of 0.44 ± 0.21 kPa in white matter and 0.23 ± 0.07 kPa in gray matter indicated through the gray boxes.

**Fig. 5.**
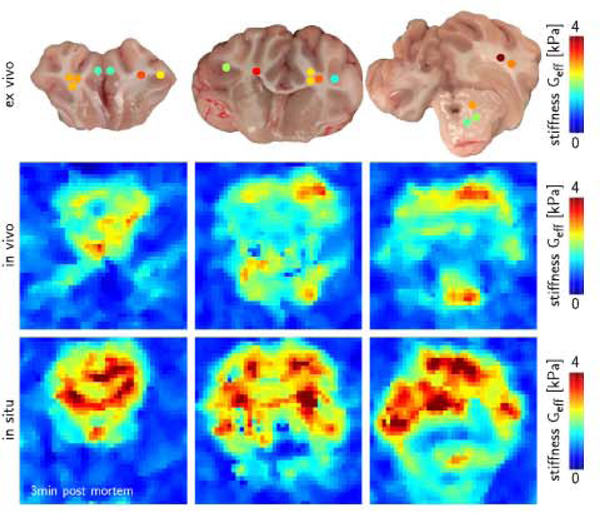
In vivo, in situ, and ex vivo effective stiffnesses $G_{\text{eff}}={\left[G^{\prime 2}+G^{\prime \prime 2}\right]}^{1/2}$ from magnetic resonance elastography and nanoindentation across three representative coronal slices at an activation frequency of 80 Hz. Maximum effective stiffnesses were 3.7 kPa in vivo, 4.5 kPa in situ 3 min post mortem, and 3.9 kPa ex vivo. White matter was generally stiffer than gray matter and displayed notable stiffness variations, both in vivo and ex vivo.

Fig. 5 compares the in vivo, in situ, and ex vivo effective stiffnesses $G_{\text{eff}}={\left[G^{\prime 2}+G^{\prime \prime 2}\right]}^{1/2}$ from magnetic resonance elastography and nanoindentation across three representative coronal slices at an activation frequency of 80 Hz. For a direct comparison, we measured the exact location of our three coronal slices prepared for nanoindentation and manually selected the three elastography slices that best matched these three indentation slices. Their in vivo, in situ, and ex vivo recordings display an excellent qualitative and quantitative agreement. First, maximum effective stiffnesses were of similar magnitude, 3.7 kPa in vivo, 4.5 kPa in situ, and 3.9 kPa ex vivo. Second, white matter was generally stiffer than gray matter in all three cases, which also agrees with the data reported in Table 1. Third, white matter displayed notable regional stiffness variations, in vivo, in situ, and ex vivo.

### Ex vivo white matter stiffness increases with myelin content

**Fig. 6.**
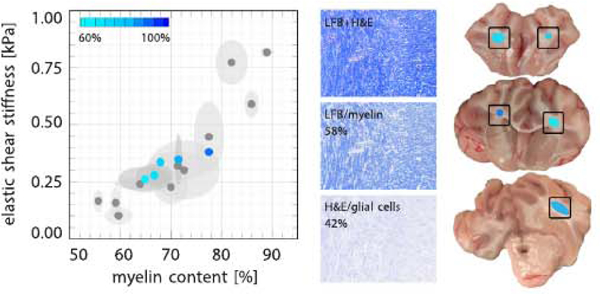
Ex vivo elastic shear stiffness from nanoindentation versus local myelin content from histochemical analysis. Stiffness increases with myelin content. Elastic shear moduli ranged from 0.28 to 0.38 kPa; myelin fractions ranged from 67 to 78%. Blue points indicate the mean, ellipses indicate standard deviations. Gray points, for comparison, indicate measurements from our previous studies (Weickenmeier et al., 2016, Weickenmeier et al., 2017). The histochemical stains illustrate the color separation into myelin (blue) and glial cells (purple). The three coronal slices show the regions associated with the five data points in the graph.

Fig. 6 highlights the correlation between the ex vivo elastic shear stiffness and the local myelin content After nanoindentation, we excised the indented regions and we performed histochemical analyses using hemotoxylin and eosin and luxol fast blue staining. We post-processed the images via color separation into myelin content, shown in blue, and glial cells, shown in purple. The three coronal slices show five representative regions associated with the five blue data points in the graph. Each blue data point indicates the mean, and its ellipse indicates the standard deviations. Gray points, for comparison, indicate measurements from our previous studies (Weickenmeier et al., 2016, 2017). White matter stiffness increased with increasing myelin content. The elastic shear moduli ranged from 0.28 to 0.38 kPa and the corresponding myelin fractions ranged from 67 to 78%.

### The standard linear solid model provides the best fit

**Fig. 7.**
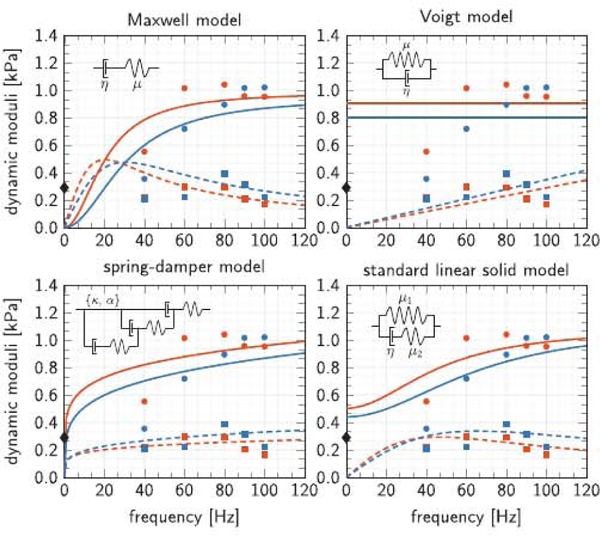
Viscoelastic models. In vivo storage and loss moduli of the cerebrum (red) and cerebellum (blue) and best fit Maxwell model, Voigt model, spring-damper model, and standard linear solid model. 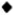 ex vivo quasi-static shear modulus from nanoindentation of 0.27 kPa; 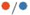 in vivo storage moduli from magnetic resonance elastography; 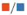 in vivo loss moduli from magnetic resonance elastography; 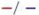 model-based storage moduli; 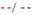 model-based loss moduli.

Fig. 7 illustrates the in vivo storage and loss moduli measured through magnetic resonance elastography in the cerebrum, shown in red, and the cerebellum, shown in blue, and the best fit of four commonly used viscoelastic models, the Maxwell model, Voigt model, spring-damper model, and standard linear solid model. The black diamond illustrates the ex vivo quasi-static shear modulus from nanoindentation of 0.27 kPa. The red and blue circles and squares highlight the mean in vivo storage and loss moduli from magnetic resonance elastography according to Fig. 2. These measurement points are the same across all four graphs. The red and blue solid and dashed lines show the storage and loss moduli predicted by the four viscoelastic models. Notably, the Maxwell model and the spring-damper model both predict a non-physical zero storage modulus in the quasistatic limit at zero frequency, whereas the Voigt model and the standard linear solid model both predict a non-zero storage modulus in the quasi-static limit The Voigt model predicts a constant, frequency-independent storage modulus, whereas the standard linear solid model predicts an increasing storage modulus with increasing frequency up to a characteristic plateau value in the infinite-frequency limit.

Table 2 summarizes the rheological models, the functional relations, and the viscoelastic model parameters of the Maxwell model, Voigt model, spring-damper model, and standard linear solid model. The viscoelastic parameters are a result of minimizing the normalized root mean square errors between the experimentally measured storage and loss moduli and the moduli predicted by the four models. The table reports the region-specific model parameters and errors for all five regions, the cerebrum, cerebellum, corpus callosum, thalamus, and brainstem. Of all four models, only the Voigt model and the standard linear solid model predict a non-zero shear stiffness *μ* in the quasi-static limit: For the Voigt model, it ranges from 0.80 to 1.01 kPa, which is about three times the quasi-static shear stiffness of 0.27 kPa; for the standard linear solid model, it ranges from 0.43 to 0.61 kPa, which is about twice the quasi-static shear stiffness of 0.27 kPa. The normalized root mean square error allows for a comparison between the individual models. The error between experiment and model varies from 6 to 28%. The Voigt model with an average error of 17% provided the worst fit, primarily because of its constant, frequency-independent elastic stiffness modulus, which overestimates the storage modulus at lower frequencies. Notably, the standard linear solid model with an average error of 11% provided the best fit.

## 4. Discussion

### Magnetic resonance elastography is a non-invasive and regionally specific measurement technique

Previous techniques to characterize brain stiffness in vivo are based on direct contact measurements through tissue indentation. Those techniques are invasive, they require a partial removal of the skull, and can only record a single measurement at a time (Miller et al., 2000; Gefen and Margulies, 2004; Prevost et al., 2011). It is well understood today that the central nervous system is a highly dynamic system with a tightly regulated perfusion (Drew et al., 2011), oxygenation, and rapid fluid turnover to maintain its high metabolic rate (Tsurugizawa et al., 2013; Thrane et al., 2014). Removing regions of the skull for mechanical testing changes the intracranial pressure and affects the natural equilibrium state, while elastography inherently preserves the in vivo equilibrium. It is thus not surprising that some reported stiffness values from indentation vary significantly, partly also due to differences in animal models, loading conditions, indenter shape, and preconditioning (Vappou et al., 2007). Another obvious disadvantage of in vivo indentation is that it only records a single averaged tissue stiffness at a time while elastography provides a three-dimensional stiffness map across the entire brain.

Fig. 2 highlights noticeable stiffness variations across the brain, where myelin-dense regions such as the corpus callosum and brainstem are generally stiffer than the cerebral average. Our in vivo elastography stiffnesses of the porcine brain are, on average, half as stiff as measurements reported for human brain (Testu et al., 2017; Sack et al., 2008; Weickenmeier et al., 2018) and an order of magnitude softer than the brains of mice(Atay etal., 2008; Murphy et al., 2011; Clayton etal., 2011), rats (Vappou et al., 2008; Shulyakov et al., 2009), and ferrets (Feng et al., 2013b). Human brains display similar micro- and macrostructural features as porcine brains including size, gyrification, and tissue composition, which results in comparable brain stiffnesses (Weickenmeier et al., 2018). Mouse and rat brains, however, are notably smaller and less folded which could explain their higher stiffnesses. As a natural consequence, their optimal activation frequencies can be up to one order of magnitude larger than those of porcine and human brains (Clayton et al., 2011).

### Brain tissue stiffens significantly immediately post mortem

Mechanical testing of brain tissue is typically performed ex vivo with various post mortem times. While we have previously shown that the brain tissue stiffness varies less than 5% within two hours and five days post mortem (Budday et al., 2015), no existing study has reported stiffness variations immediately after death. Within only three minutes post mortem, we observed a stiffening of 26% in the cerebrum, and an even more pronounced stiffening of 58% in the corpus callosum. In response to emerging states of hypoxia and ischemia, neuronal and astroglial cells instantaneously adapt and initiate extensive compensatory mechanisms to delay neuronal damage. Traumatic and ischemic brain injuries may–and death certainly will–trigger depolarizing waves that propagate through the brain at propagation speeds of 2–5 mm/min and lead to a complete depolarization of cellular membranes (Santos et al., 2014). The collapse of the ionic homeostasis in the dying brain leads to a substantial influx of water (Sevick et al., 1992) and sub-sequent neuronal and astroglial swelling (Risher et al., 2012). These changes occur within minutes of traumatic injury and, if sustained, lead to comprehensive neuronal cell death due to cytotoxic edema (Rungta et al., 2015). Cytotoxic edema is generally accompanied by energy depletion, a phenomenon associated with enhanced influx of glymphatic cerebral spinal fluid and suppressed efflux of interstitial fluid from the cranium. Within short periods of time, the resulting fluid build-up leads to a noticeable increase in intracranial pressure (Thrane et al., 2014) and tissue stiffening (Vappou et al., 2008).

Fig. 3 and Table 1 reveal an immediate increase in storage and loss moduli, which agree well with these cellular mechanisms: While regions with a high cell density show a pronounced post mortem stiffening, regions with a low neuronal cell density, such as the brainstem, stiffen by less than 8%. While small animal studies cannot resolve different brain regions as accurately as our current pig study, they can still provide general insight into the mechanical properties post mortem: Elastography studies of mice brain found no significant change in storage moduli 2 h post mortem (Atay et al., 2008) whereas studies of rat brain observed a 100% increase in storage moduli 30min post mortem followed by a drop to 18% above the initial value at 24 h post mortem (Vappou et al., 2008). The loss modulus also increased by 28% within 30min and subsequently dropped to only 73% of the in vivo loss modulus within 24 h post mortem. This agrees well with our observations of an immediate brain stiffening 3 min post mortem. In addition, our study also found a continuing brain stiffening within the first 45 min post mortem and a significant regional variation of these stiffening effects. In contrast to our elastography recordings, previous indentation measurements did not display statistical differences between the mechanical characteristics in vivo and in situ and did also not reveal regional variations (Gefen and Margulies, 2004). However, those indentations only probe the cortical surface whereas our study records mechanical characteristics across the entire brain. Our study agrees well with a recent study based on in vivo, in situ, and in vitro indentation through a cranial window, which reported a significant stiffening in situ compared to in vivo and followed by a significant softening ex vivo compared to in situ (Prevost et al., 2011). Notably, mechanical tissue changes after death appear across all spatial and temporal scales and, as the literature shows, their characterization is highly sensitive to various experimental factors including storage conditions, storage time, and testing method.

### Regional stiffness variations remain present post mortem

Fig. 4 summarizes the ex vivo storage and loss moduli for frequencies between 10 and 100 Hz and the elastic shear moduli from nanoindentation within 16 h post mortem. When directly comparing these ex vivo indentation recordings to in vivo elastography measurements, we have to keep in mind that elastography measures the stiffness of the intact tissue whereas indentation is performed on tissue slices. We know that the living brain, like many other soft tissues in our body, is exposed to pre-strain and residual stresses in vivo (Rausch et al., 2013; Buganza Tepole et al., 2016) and that slicing the tissue alters its mechanical environment (Xu et al., 2010). While preparing tissue slices for indentation naturally changes the mechanics of the sample, we observed that these effects are less drastic than the changes upon cutting small cubical samples for mechanical testing (Budday et al., 2017c). We have previously shown that flat-punch indentation on brain slices is a robust, reliable, and repeatable method to characterize ultrasoft gray and white matter tissue (Budday et al., 2015). Our recorded moduli lie well within the range of values reported in literature (Elkin et al., 2011a; Forte et al., 2017; Chen et al., 2015; van Dommelen et al., 2010; Lu et al., 2006; Prevost et al., 2011; Samadi-Dooki et al., 2017). In agreement with the literature, our loss modulus increases for increasing frequencies and exceeds the magnitude of the storage modulus (Samadi-Dooki et al., 2017). Several studies suggest to correct these moduli by a correction factor (Hayes et al., 1972; Finan et al., 2014), in our case ~1.2, to account for our large probe-radius-to-sample-thickness ratio of 0.75 mm/5 mm. This correction factor can be determined via finite element simulations (Samadi-Dooki etal., 2018) and depends on the contact condition between the brain slice and the petri dish. Here we observed no-slip boundary conditions between the slice and the dish and we confirmed that our contact stiffness scales linearly with our probe radius, from 0.375 mm to the radius of 0.750 mm used here (Budday et al., 2015). We thus decided to not correct our measurements. While our elastic shear moduli in Fig. 4, right, reliably and reproducibly match previously reported values (Budday et al., 2015; Weickenmeier et al., 2016, 2017), our storage and loss moduli in Fig. 4, left, might be limited by the amplitude of our testing device. Ideally, for a flat punch with a diameter of *d* = 1.5 mm, an average strain of (4 δ)*/*(*π d*) for flat punch indentation (Elkin et al., 2011b), and a linear strain regime of 1% (Nicolle et al., 2004; Feng et al., 2013a), we should use an indentation depth of *δ* = 11 μm. Our selected indentation amplitude is close to the transducer’s sensitivity limit and might result in a response that is sensitive to surface effects such as hydrophilic adhesion between the tip and the tissue.

Fig. 5 confirms a good quantitative agreement between the in vivo, in situ, and ex vivo effective stiffnesses from magnetic resonance elastography and nanoindentation. The effective stiffness across all three coronal slices matches both in magnitude and in regional variation; in fact, the inner dense white matter regions are noticeably stiffer than the outer cortex (Weickenmeier et al., 2016). Overall, our in vivo, in situ, and ex vivo recordings agree well with the stiffness values of other studies (Vappou et al., 2008; Prevost et al., 2011; Gefen and Margulies, 2004); yet, despite all efforts, it remains unknown how precisely the in situ brain characteristics evolve longitudinally post mortem and how excising the brain affects these stiffness alterations.

Fig. 6 highlights the correlation between the white matter stiffness and the local myelin content, which may, at least in part, explain these regional stiffness variations. Our current measurements, highlighted in blue, agree well with previous histological analyses shown in gray for comparison (Weickenmeier et al., 2016, 2017). The observed correlation between stiffness and myelination agrees well with the stiffness reduction observed upon controlled demyelination in a multiple sclerosis model in mice (Schregel et al., 2012).

### Brain stiffness and viscosity are 1.0 kPa and 0.01 kPa s

Our results agree with previous observations that the in vivo storage and loss moduli from magnetic resonance elastography strongly depend on the activation frequency (Clayton et al., 2011). To convert the frequency-dependent storage and loss modulus into frequency-independent viscoelastic parameters, we identified the parameters of four common viscoelastic models, the Maxwell and Voigt models, the spring-damper model, and the standard linear solid model (Sack et al., 2009). While it is well understood that brain tissue is nonlinear (Finan et al., 2017), structurally anisotropic (Anderson et al., 2016; Feng et al., 2013a), and locally specific (Budday et al., 2017a), our linear viscoelastic parameters in Table 2 provide a reasonable first approximation of the viscoelastic behavior of different brain regions; they can be easily compared to previous measurements and directly used for linear finite element simulations.

Pigs have been identified as useful animal models for trauma and neurodegenerative disease given their similarities with human brains (Jean et al., 2014; Eaton and Wishart, 2017). In general, our recorded values agree well with the reported viscoelastic elastography moduli of human brain tissue (Klatt et al., 2007; Sack et al., 2009; Testu et al., 2017). Human brain moduli, averaged over the entire brain, were *μ =* 2.3 kPa and *η =* 0.015 kPa s for the Maxwell model, *μ* = 1.8 kPa and *η =* 0.003 kPa s for the Voigt model, and *μ*_1_, = 0.8 kPa, *μ*_2_ = 2.0 kPa, and *η* = 0.007 kPa s for the standard linear solid model (Klatt et al., 2007). Another study found human brain moduli of *μ =* 1.8 kPa and *η* = 0.015 kPa s for the Maxwell model, *μ =* 1.5 kPa and *η* = 0.002 kPa s for the Voigt model, and *μ*_1_ = 1.0 kPa, *μ*_2_ *=* 1.5 kPa, and *η* = 0.0039 kPa s for the standard linear solid model (Sack et al., 2009). While the shear stiffness *μ,* on the order of kilopascals from in vivo elastography generally agrees well with shear stiffnesses from ex vivo indentation (Samadi-Dooki et al., 2018) and triaxial testing (Budday et al., 2017c), the relaxation constants *η* can vary by up to three orders of magnitude from pascal second recorded during in vivo elastography (Klatt et al., 2007; Sack et al., 2009; Testu et al., 2017; Weickenmeier et al., 2018) to kilopascal second recorded during ex vivo indentation (Samadi-Dooki et al., 2018) and triaxial testing (Budday et al., 2017a; Budday et al., 2017b). Clearly, further studies are needed to explain the mechanistic origin of these discrepancies.

Fig. 7 and Table 2 suggest that the standard linear solid model, with a finite storage modulus in the quasi-static limit, is best suited to characterize the viscoelastic behavior of brain tissue. In all five brain regions, its shear stiffness *μ = μ*_1_ +*μ*_1_ was on the order of 1.0 kPa, similar to the shear stiffness *μ* of the Maxwell and Voigt models, and its viscosity *η* was on the order of 0.01 kPa s. Our quasi-static shear modulus for the cerebrum is similar to data for porcine (Prange and Margulies, 2002; Gefen and Margulies, 2004) and human brain (Budday et al., 2017a). Several recent studies report regional variations of constitutive moduli (Prange and Margulies, 2002; Johnson et al., 2013); yet, the collective analysis across all studies fails to reveal a consistent trend (Budday et al., 2017c; Weickenmeier et al., 2016).

### Limitations

Our study has several limitations that we have to keep in mind when interpreting the data. First, our findings are currently based on the observations of a single porcine brain; yet, data reported here are in line with previous findings in mouse (Clayton et al., 2011), ferret (Feng et al., 2013a), and human (Sack et al., 2009) and the intra-subject variation among different testing methods is consistent with our previous work (Weickenmeier et al., 2016, 2017). Second, despite many structural similarities between human and porcine brains, differences in skull thickness, brain volume, and brain anatomy can cause deviations in elastography measurements between pig and human (Weickenmeier et al., 2018). Third, based on the heterogeneous nature of white matter tissue, small variation in location can result in large changes in mechanical properties (Weickenmeier et al., 2016), which may not only limit the direct comparison between ex vivo slices and in vivo elastography, but also the interpretation of the elastography recordings as a whole. Fourth, although our longitudinal in vivo, in situ, and ex vivo study provides a comprehensive understanding of tissue property changes with time, these changes have yet to be connected to the underlying biochemical processes to identify the mechanistic origin of change. In addition to increasing the number of animals to build confidence in the method, our future studies will include longitudinal histology to better understand the time line of tissue degeneration post mortem. As a first step, our future work will focus on characterizing the range of stiffness fluctuation in the healthy human brain in vivo with varying perfusion and intracranial pressure.

## 5. Conclusion

We characterized the in vivo, in situ, and ex vivo properties of five different brain regions for four common viscoelastic models, the Maxwell, Voigt, spring-damper, and standard linear solid model. For the first time, we performed non-invasive, in vivo and in situ elastography and ex vivo indentation on one and the same brain to characterize viscoelastic properties under different metabolic conditions. Strikingly, we observed an immediate brain stiffening of up to 58% within only three minutes post mortem and a continuing stiffening of up to 142% within 45 minutes. Our results suggest that only in vivo measurements can provide a true rheological picture of the brain: Ex vivo measurements severely *overestimate* the stiffness of the brain and *underestimate* injury risk in response to physical forces. Similar to many other organ systems, the living brain is exposed to a tightly regulated mechanical environment in vivo that changes drastically when tissue pieces are cut for mechanical testing and pre-strain and residual stress are released. In addition, unlike most parts of our body, our brain experiences significant changes in polarization, oxidation, perfusion, and metabolism immediately after injury or death. While most existing studies assume that studying the brain ex vivo provides sufficient insight into its mechanical behavior, we are only beginning to understand the important role of metabolic activity within the biophysics of the human brain. Characterizing the true in vivo environment of the brain has important consequences in understanding the physical biology of the brain, designing neurodevices, planning neurosurgery, and interpreting neurodegeneration.

## Figures and Tables

**Table 1:** In vivo, in situ, and ex vivo storage and loss moduli for five brain regions at an activation frequency of 80 Hz. Storage and loss moduli increase with post mortem time. Stiffening within 3min post mortem was 58% in the corpus callosum, followed by 38% in the thalamus, 26% in the cerebrum, 17% in the cerebellum, and 8% in the brainstem. Within 45 min, post mortem stiffening continued. For comparison, within 16 h post mortem, moduli of a comparison brain were more than twice as stiff.

storage modulus [kPa]					
region	in vivo	in situ 3 min pm	in situ 45 min pm	ex vivo 16 h pm	in situ 16 h pm

**cerebrum**	1.13	1.42	1.58	1.52	2.60
**cerebellum**	0.88	1.03	1.29	–	2.36
**corpus call**	1.22	1.93	2.95	–	4.57
**thalamus**	1.14	1.57	1.84	–	2.59
**brainstem**	1.01	1.09	1.08	–	1.49

loss modulus [kPa]					

region	in vivo	in situ 3 min pm	in situ 45 min pm	ex vivo 16 h pm	in situ 16 h pm

**cerebrum**	0.30	0.48	0.61	2.36	1.02
**cerebellum**	0.33	0.48	0.56	–	0.95
**corpus call**	0.43	0.76	1.14	–	2.26
**thalamus**	0.32	0.59	1.30	–	1.69
**brainstem**	0.20	0.19	0.30	–	0.39

**Table 2:** Viscoelastic models. Rheological models, functional relations, and parameters of the Maxwell model, Voigt model, spring-damper model, and standard linear solid model determined from frequency-dependent in vivo storage and loss moduli.